# Cecal Intussusception Diagnosed by Total Colonoscopy in a Child: A Case Report

**DOI:** 10.3389/fped.2020.00438

**Published:** 2020-08-04

**Authors:** Toshihiko Kakiuchi, Motohiro Esaki, Aiko Nakayama, Fumio Ichinose, Muneaki Matsuo

**Affiliations:** ^1^Department of Pediatrics, Faculty of Medicine, Saga University, Saga, Japan; ^2^Division of Gastroenterology, Department of Internal Medicine, Faculty of Medicine, Saga University, Saga, Japan

**Keywords:** intussusception, total colonoscopy, child, lead point, computed tomography

## Abstract

**Background:** Ileocolic intussusception is the most common form of intussusception in children. Intussusception in the appendix or cecum without a lead point in a child is very rare and was found with total colonoscopy (TCS) and computed tomography.

**Case Presentation:** A 9 year-old boy was admitted to our hospital with fever, vomiting, and two episodes of bloody diarrhea. Inflammatory bowel disease was suspected; TCS was performed and revealed intussusception whose advanced region was in the cecum. The diagnosis was idiopathic cecum intussusception. This case was unusual in that intussusception had occurred at a young age but without lead point; in addition, the intussusception had also occurred at the tip of the cecum. The intussusception was safely reduced by endoscopic procedures, and after improvement in the vomiting, the patient was safely discharged and has had no bloody stools since.

**Conclusion:** We demonstrated cecal intussusception without lead point observed on TCS in a child.

## Introduction

Intussusception refers to the invagination of a part of the intestine into itself. Intussusception is classified by location; ileocolic intussusception involves the ileocecal junction and accounts for 90% of all pediatric cases of intussusception (1). Approximately 60% children with intussusception are younger than 1 year, and 80–90% are younger than 2 years (1–3). In ~25% cases, an underlying disease causes the development of a pathologic lead point for the intussusception. Such triggers account for a greater proportion of cases of intussusception in children younger than 3 months or older than 5 years (4, 5).

In adults, most cases of appendiceal or cecal intussusceptions occur with lead points, and surgery is the choice of treatment. Therefore, endoscopic examination is rarely performed. In contrast, appendiceal or cecal intussusception without a lead point is very rare, especially in children (6). We report a case of idiopathic cecal intussusception diagnosed in a child during total colonoscopy (TCS).

## Case Presentation

A 9 year-old boy was admitted to our hospital with fever, vomiting, and bloody diarrhea. He had suffered cardiopulmonary arrest and hypoxic encephalopathy caused by a subdural hematoma that resulted from abuse immediately after birth and had been managed at home with a ventilator and tube nutrition. He was usually bedridden and unable to communicate with the caregiver at all. On his initial visit to our hospital, his vital signs were abnormal: Body temperature was 39.2°C, heart rate was 153 beats per minute, and saturation of percutaneous oxygen was 90% without supplemental oxygen. A physical examination revealed no abnormal findings in his chest or abdomen. His height was 106 cm (standard deviation, 4.5 cm), and weight was 22.6 kg (standard deviation, 1.1 kg). His white blood cell count was 13,300/μL (normal range, 3,300–8,600), and the proportion of neutrophils was 79.6% (normal range, 40–69%). Serum C-reactive protein level (3.74 mg/dL; normal, <0.14 mg/dL) and erythrocyte sedimentation rate (20.0 mm/h; normal, 3.0–15.0 mm/h) were elevated (Table 1). Stool bacterial cultures did not reveal pathological bacteria. Rapid antigen tests for rotavirus, adenovirus, and norovirus yielded negative results. The fecal calprotectin level was 2,420 mg/kg (normal, <300 mg/kg). Chest and abdominal plain radiographs showed no obvious abnormalities other than severe scoliosis. His diagnosis was acute bacterial gastroenteritis, and hydration and intravenous antibiotics (ampicillin, 1 g, three times a day) were started. On the fourth day of treatment, his temperature went down and the vomiting and diarrhea disappeared, and he was allowed to leave the hospital.

**Table 1:** Blood test and biochemical test results at the first visit.

WBC	13,300	/μL	TP	8.6	g/dL
Neu	79.6	%	Alb	4.7	g/dL
Lym	16.3	%	AST	20	U/L
Mo	3.3	%	ALT	20	U/L
Hb	18.3	g/dL	LDH	276	U/L
Plt	490 × 10^3^	/μL	BUN	16.8	mg/dL
			Cre	0.49	mg/dL
			Na	148	mEq/L
PT-INR	1.15		K	3.5	mEq/L
APTT	32.4	sec	Cl	102	mEq/L
Fib	551.4	mg/dL	CRP	3.74	mg/dL
			ESR	20	mm/H

*WBC, white blood cell; Neu, neutrophil; Lym, lymphocytes; Mo, monocyte; Hb, hemoglobin; Plt, platelet; PT-INR, international normalized ratio of prothrombin time; APTT, activated partial thromboplastin time; Fib, fibrinogen; TP, total protein,; Alb, albumin; AST, aspartate transaminase; ALT, alanine aminotransferase; LDH, lactate dehydrogenase; BUN, blood urea nitrogen; Cre, creatinine; Na, natrium; K, kalium; Cl, chloride; CRP, C-reactive protein; ESR, erythrocyte sedimentation rate*.

**Figure 1:**
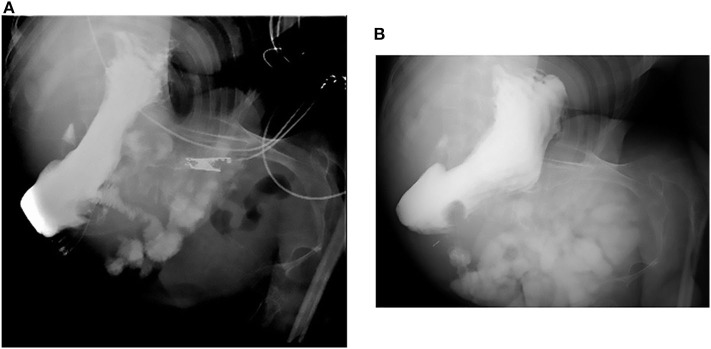
Fluoroscopy. **(A,B)** The upper gastrointestinal tract did not reveal any obvious abnormal findings, and the C-loop was also formed normally.

Five days after discharge, fever and vomiting recurred, and he had persistent bloody stools, similar to those at the time of earlier admission. His vital signs were abnormal: body temperature was 37.3°C, and heart rate was 153 beats per minute. Physical examination revealed no abnormal findings. His white blood cell count was 10,300/μL, and the proportion of neutrophils was 77.0%. Serum C-reactive protein level was elevated (7.19 mg/dL), as was the erythrocyte sedimentation rate (20.0 mm/h; Table 2). Abdominal plain radiographs showed stomach bubble expansion and intestinal tract dilation, but the chest radiograph showed no abnormalities. Fluoroscopy with amidotrizoate to confirm passage through the upper gastrointestinal tract did not reveal any obvious abnormal findings and revealed a C-loop after the descending pedicle of the duodenum (Figures 1A,B).

**Table 2:** Blood test and biochemical test results at the second visit.

WBC	10,300	/μL	TP	8.6	g/dL
Neu	77.0	%	Alb	4.4	g/dL
Lym	16.5	%	AST	15	U/L
Mo	4.5	%	ALT	25	U/L
Hb	18.3	g/dL	LDH	329	U/L
Plt	558 × 10^3^	/μL	BUN	27.8	mg/dL
			Cre	0.39	mg/dL
			Na	146	mEq/L
PT-INR	1.08		K	3.7	mEq/L
APTT	32.2	sec	Cl	99	mEq/L
Fib	931.6	mg/dL	CRP	7.19	mg/dL
			ESR	20	mm/H

*WBC, white blood cell; Neu, neutrophil; Lym, lymphocytes; Mo, monocyte; Hb, hemoglobin; Plt, platelet; PT-INR, international normalized ratio of prothrombin time; APTT, activated partial thromboplastin time; Fib, fibrinogen; TP, total protein,; Alb, albumin; AST, aspartate transaminase; ALT, alanine aminotransferase; LDH, lactate dehydrogenase; BUN, blood urea nitrogen; Cre, creatinine; Na, natrium; K, kalium; Cl, chloride; CRP, C-reactive protein; ESR, erythrocyte sedimentation rate*.

**Figure 2:**
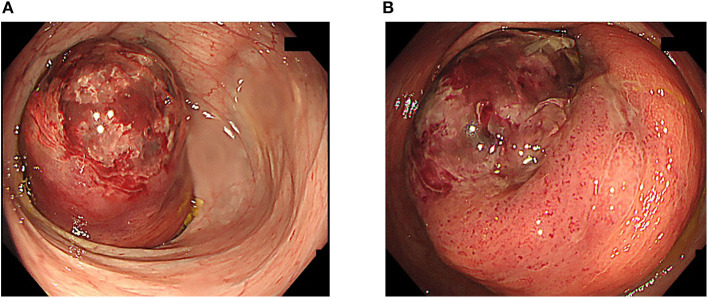
Total colonoscopy. **(A)** Total colonoscopy showed intussusception of the intestine with an ulcer at the top at the transverse colon. **(B)** The tip of the intussusception was red and had formed an ulcer.

**Figure 3:**
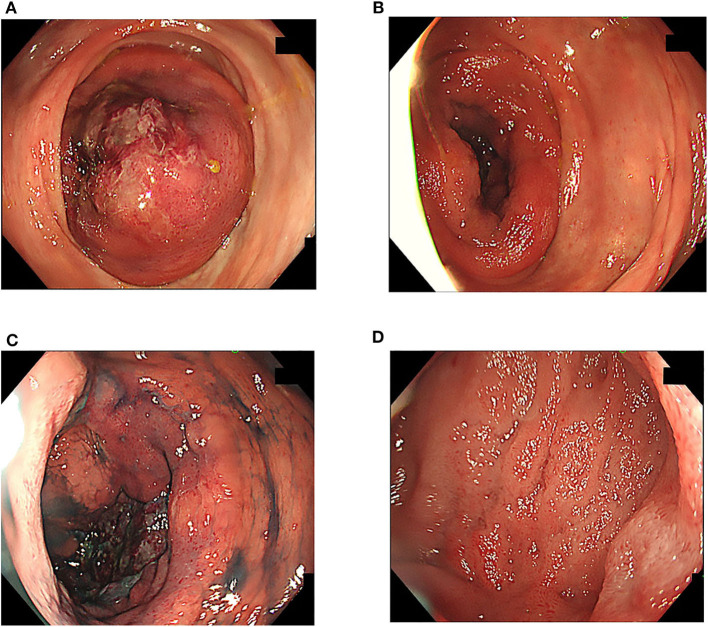
Total colonoscopy. **(A,B)** The intussusception was gradually reduced with endoscopic insufflation. **(C)** Ulcer formation was observed in the cecum, but no neoplastic lesion was observed. **(D)** Almost no abnormal findings were found between the ileocecum and the distal ileum.

**Figure 4:**
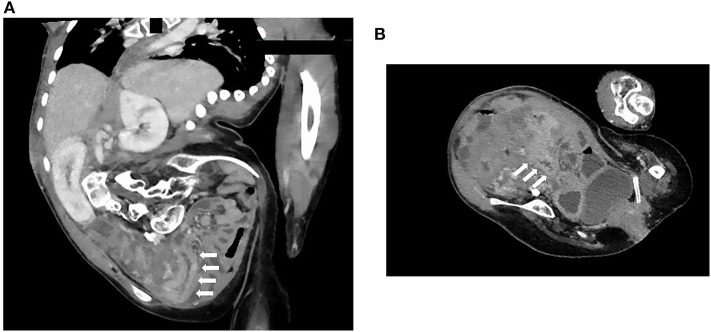
Abdominal contrast medium-enhanced computed tomography. **(A,B)** The images revealed an effect of enhancing the mucosal surface and the edematous wall thickening localized from the terminal ileum to the cecum. White arrows indicate the terminal ileum and cecum.

TCS was performed because we suspected inflammatory bowel disease on the basis of persistent bloody stool, increased inflammatory response in blood tests, and elevated fecal calprotectin levels. However, TCS demonstrated intussuscepted intestine with an ulcer on top in the transverse colon (Figures 2A,B). We thus reduced the intussusception by endoscopic manipulation and insufflation (Figures 3A,B). After the reduction, the ulcer was observed in the cecum, but no neoplastic lesion that precipitated intussusception was noted (Figure 3C). Appendiceal orifice could not be clearly detected because of the cecal ulceration and reddish edematous mucosa; however, no other abnormal finding was observed from the distal ileum to the cecum (Figure 3D). Biopsy was not performed on TCS procedure because we were worried about intestinal perforation. Contrast medium-enhanced abdominal computed tomography (CT) subsequently performed on the next day revealed enhanced mucosal surface and edematous wall thickening from the terminal ileum to the cecum (Figures 4A,B). We performed CT scan that could be performed in a shorter time than an MRI scan, because the patient was on a respiratory ventilator. We thus diagnosed the condition as cecal intussusception and the cecum became the advanced part, where ulceration could be caused by mechanical stimulation and ischemic changes. After improvement in intussusception, his clinical symptoms promptly disappeared, and the patient was safely discharged home and has had no bloody stools since.

## Discussion

This report presents an idiopathic cecal intussusception diagnosed by TCS in a child. The notable characteristic of this case was intussusception at a young age (9 years), with no lead point, which occurred at the tip of the cecum. In addition, we were able to observe intussusception on TCS.

Ileocolic intussusception is the most common form of intussusception in children (7). The development of intussusception may result from the invagination of the muscular ileocecal valve into the cecum because the cecal wall is not rigid, as a result of the paucity of developed taeniae coli (8). In our patient, after intussusception was reduced by TCS, ulceration and mucosal edema were observed throughout the cecum, but we found almost no abnormalities in the terminal ileum. We thus judged the lead point to be the cecum. In adults, cecal intussusception based on cecal cancer (9), lipoma (10), mobile cecum (11), and double intestinal tract (12) has been reported; however, no such findings have been reported so far in children. Such a discordant result might be partly attributed to the fact that endoscopy is rarely performed in children. It can be possible that cases of pediatric intussusception are classified as ileocolic intussusception without further investigation, except during surgery.

Pediatric intussusception is usually idiopathic; in only 10% of cases is a precipitating lesion identifiable (13). The sensitivity and specificity of ultrasonography in the diagnosis of intussusception, especially in children, approach nearly 100% when it is performed by experienced clinicians. Because of its non-invasive nature, ultrasonography is the imaging modality of choice for evaluating children and is useful for prompt screening of intussusception (14). Therefore, TCS is rarely performed in children with intussusception. Tajik et al. reported a 28 month-old boy in whom ileocolic intussusception was diagnosed on TCS (15). Ileocolic intussusception, the most common type in children, requires reduction by ultrasound-guided or fluoroscopic pneumatic or hydrostatic enema, which is successful in 85–90% of cases (16). The present case demonstrated that TCS enabled the diagnosis, and intussusception was safely reduced endoscopically. Tafner et al. (17) reported that 20 of 30 pediatric intussusception patients could be safely reduced by colonoscopy, and this method is safe because it is non-invasive and the intestine can be directly observed. On the other hand, it has been reported that intestinal intussusception develops iatrogenically after TCS, and further investigation is required for the reduction in intussusception on TCS (18–20).

In our patient, two problems remained unsolved. First, the cause of intussusception was not identified. Because laparotomy was not performed in this case, the presence of a moving cecum could not be evaluated as a cause of intussusception. In addition, he had severe scoliosis; therefore, CT could not depict intestinal malrotation (21). However, upper gastrointestinal fluoroscopy revealed a C-loop after the descending pedicle of the duodenum, suggesting a low possibility of intestinal malrotation. Biopsy was not performed from the ulcer of the cecum because of the fear of intestinal perforation at the time of TCS, and our judgment was only a macroscopic evaluation. Second, TCS did not clearly depict appendiceal orifice, and the appendix was not completely evaluated during CT; therefore, we could not rule out the possibility of appendiceal intussusception. Further, we did not perform CT or ultrasonography before TCS because not intussusception but inflammatory bowel disease was suspected because of the inflammatory findings and long-lasting bloody stools. It was because we were worried that we could not understand the location of the intestine due to strong scoliosis; we should reflect on this in clinical setting henceforth.

In conclusion, a 9 year-old boy was confirmed to have idiopathic cecal intussusception without a lead point on TCS, and the intussusception was safely reduced by endoscopic procedures. Based on the endoscopic findings, the cecum was considered to be the site of origin of the intussusception. Intussusception must be considered when treating patients with vomiting and fever with prolonged bloody stool symptoms.

## Data Availability Statement

The raw data supporting the conclusions of this article will be made available by the authors, without undue reservation, to any qualified researcher.

## Ethics Statement

Written informed consent was obtained from the individual(s), and minor(s)' legal guardian/next of kin, for the publication of any potentially identifiable images or data included in this article.

## Author Contributions

TK and AN contributed to the conception of the report, acquisition, analysis, interpretation of data, and helped draft the manuscript. ME and FI acquired and analyzed the data and helped draft the manuscript. MM contributed to the conception and design of the report and revised it critically for important intellectual content. All authors approved publication of the content and agreed to be accountable for all aspects of the work in ensuring that questions related to the accuracy and integrity of any part of the report are appropriately investigated and resolved.

## Conflict of Interest

The authors declare that the research was conducted in the absence of any commercial or financial relationships that could be construed as a potential conflict of interest.

## Abbreviations

CT: Computed tomography
TCS: Total colonoscopy.
